# Feasibility research of enhanced recovery after surgery implemented in esophageal cancer patients who underwent neoadjuvant chemotherapy

**DOI:** 10.1186/s12957-022-02701-3

**Published:** 2022-07-25

**Authors:** Zhanpeng Tang, Xirui Zhu, Yanzhi Li, Chenghao Qu, Lin Li, Shuhai Li, Lei Qi, Ming Lu, Chuanle Cheng, Hui Tian

**Affiliations:** Department of Thoracic Surgery, Qilu Hospital of Shandong University, Jinan, Shandong 250012 People’s Republic of China

**Keywords:** Enhanced recovery after surgery (ERAS), Neoadjuvant chemotherapy, Esophagectomy, Esophageal squamous cell carcinoma (ESCC)

## Abstract

**Background:**

Enhanced recovery after surgery (ERAS) is a perioperative management protocol to accelerate patient recovery. This study aimed to evaluate the feasibility of ERAS protocols implemented in patients who underwent neoadjuvant chemotherapy (NACT) before minimally invasive McKeown esophagectomy.

**Methods:**

This retrospective study compared the short-term clinical outcomes in esophagectomy patients from June 2018 to June 2021. Subjects were divided into two categories: those who underwent NACT (NACT group) and the non-NACT group.

**Results:**

There was no significant difference in total postoperative complication morbidity between the NACT and non-NACT groups (21.2% vs. 20.7%, *P*=0.936). In addition, the hospital length of stay post-surgery (7.90 vs. 7.71 days, *P*=0.424) was not significantly longer when compared to the non-NACT group. The time to chest tube removal (5.37 vs. 5.13 days, *P*=0.238) and first bowel movement (2.92 vs. 3.01 days, *P*=0.560) was also similar between the two groups.

**Conclusions:**

There was no significant difference in postoperative complications rate, postoperative hospital length of stay, and readmission rate between the two group. This study proved that ERAS protocols seemed to be safe and feasible for patients who received NACT before esophagectomy.

## Introduction

Among all cancers, esophageal cancer is ranked as the seventh and sixth in morbidity and mortality in the world, respectively [1]. China has a high incidence of esophageal cancer, with a rate of diagnosis that comprise between 30 and 50% of the global incidence burden [2]. The overall 5-year survival rate ranges from 15 to 25% [3]. Surgical treatment is still considered as a significant form of treatment for patients with esophageal cancer. When presenting with esophageal cancer, most patients are already locally advanced when first diagnosed. However, for most patients with locally advanced esophageal cancer, surgery alone may have a high recurrence and metastasis rates [4].

Neoadjuvant chemotherapy (NACT) has been used clinically for many years in various tumor. The purpose of NACT is to improve the rate of R0 resection for locally advanced tumor patients by preoperative chemotherapy [5].

Esophagectomy after neoadjuvant chemotherapy for esophageal cancer has been proven to be a safe and feasible method of treatment [6–8]. However, there have been no studies about the short-term clinical outcomes of esophagectomy after neoadjuvant chemotherapy for esophageal cancer patients based on enhanced recovery after surgery (ERAS) protocols. ERAS is a multidisciplinary perioperative treatment protocol which was first demonstrated by Kehlet in the late twentieth century, and the fundamental purpose of ERAS is to accelerate patient recovery [9]. The aim of this study was to investigate whether surgical treatment after neoadjuvant chemotherapy under ERAS protocols was safe and feasible for patients with esophageal cancer.

## Methods

### Patients

Qilu Hospital of Shandong University institutional review board approved this study (KYLL-202008-023). All patients signed a written informed consent to include their clinical information.

**Fig. 1:**
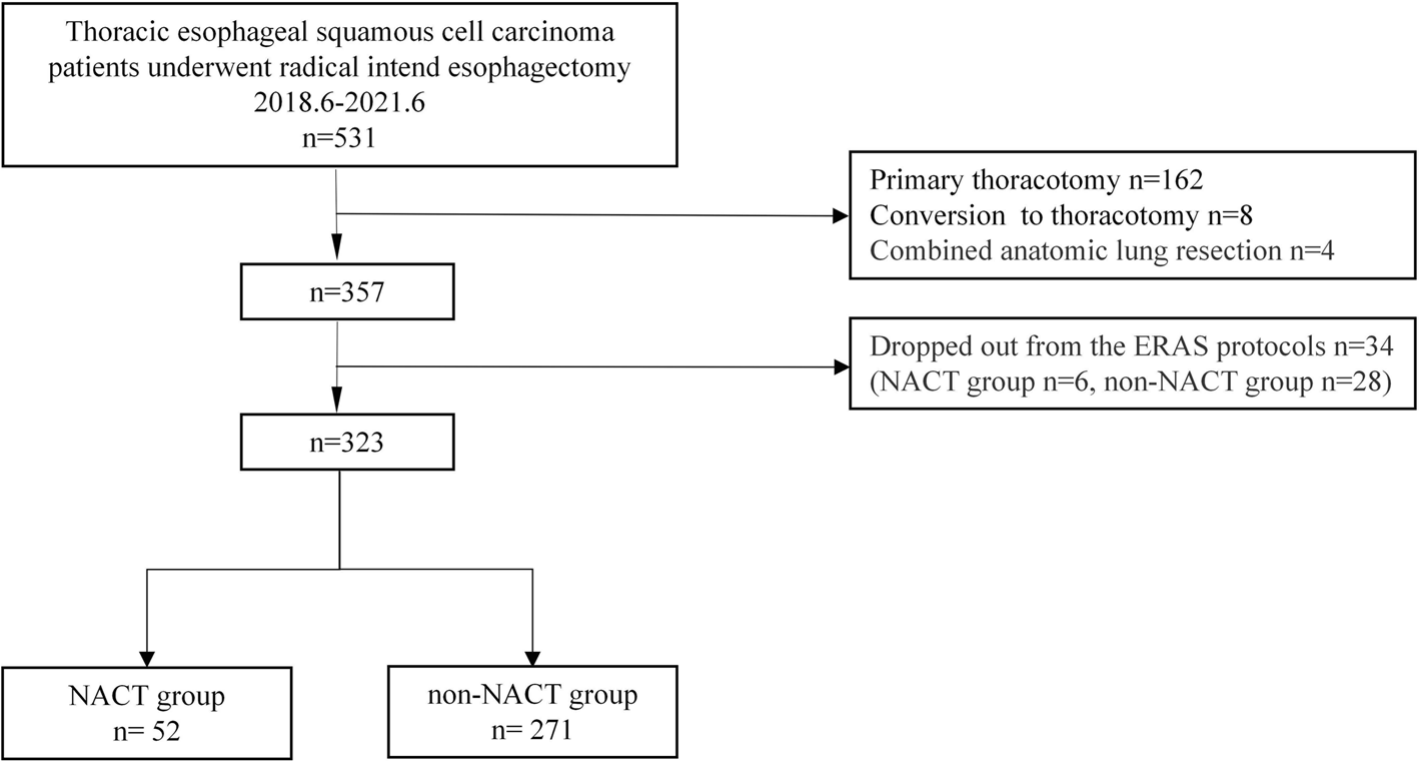
The flow diagram of the patients enrolled in this study. NACT, neoadjuvant chemotherapy

From June 2018 to June 2021, a total of 531 patients with esophageal squamous cell carcinoma (ESCC) were scheduled for esophagectomy and were treated using the ERAS protocol at the Department of Thoracic Surgery, Qilu Hospital, Cheeloo College of Medicine, Shandong University. In this study, 52 patients were treated with NACT and esophagectomy, while 271 patients were only treated with esophagectomy without any preoperative chemotherapy treatment (Fig. 1).

The inclusion criteria are shown in Table 1.

**Table 1:** Inclusion criteria

The inclusion criteria for this study	
• Adult patients (age ≥18 years) who underwent minimally invasive McKeown esophagectomy	
• Pathological diagnosis was ESCC	
• Detail medical records of patients could be allowed	
• Did not at any point stop using the ERAS protocols	

*ESCC* esophageal squamous cell carcinoma, *ERAS* enhanced recovery after surgery

### Clinical information

All the patient clinical data were collected from our hospital information system, which include the patients’ demographic, age, sex, smoking history, alcohol consumption, comorbidity (comorbidity was defined as hypertension, diabetes, coronary heart disease, and chronic diseases of digestive and respiratory system), Karnofsky Performance Status Scale (KPS), prognostic nutritional index (PNI), American Society of Anesthesiologists (ASA) physical status, tumor data, surgery-related factors, and short-term outcomes between the two groups, and are shown in Table 2. Routine blood and hepatic function tests were performed on all patients 3–5 days before surgery. Histopathologic analysis was performed by two independent pathologists. Tumor location, size, lymph node metastasis, and degrees of differentiation were recorded.

**Table 2:** Patient demographic, clinical, and operative data

Characteristic		NACT (***n***=52)	Non-NACT (***n***=271)	***P***
Age		64.35±5.77	63.59±8.54	0.542
Sex^†^	Male	45 (86.5)	222 (81.9)	0.420
	Female	7 (13.5)	49 (18.1)	
Smoking^†^	Yes	35 (67.3)	165 (60.9)	0.382
	No	17 (32.7)	106 (39.1)	
Drinking^†^	Yes	34 (65.4)	155 (57.2)	0.272
	No	18 (34.6)	116 (42.8)	
Comorbidity^†^		26 (50.0)	124 (45.8)	0.574
KPS		80.77±8.37	80.37±6.31	0.693
PNI		48.30±4.51	51.21±5.01	<.001**
ASA status^†^	I	4 (7.7)	39 (14.4)	0.264
	II	40 (76.9)	205 (75.6)	
	III	8 (15.4)	27 (10.0)	
Lymphadenectomy^†^	2D	5 (9.6)	35 (12.9)	0.508
	2D+	47 (90.4)	236 (87.1)	
Operation duration		224.10±29.58	210.76±29.21	0.003**
Estimated blood loss		121.73±33.65	111.88±42.56	0.116
Tumor location^†‡^	Upper	11 (21.2)	45 (16.6)	0.439
	Middle	20 (38.5)	111 (41.0)	
	Lower	11 (21.2)	81 (29.9)	
	Upper and middle	5 (9.6)	21 (7.7)	
	Middle and lower	5 (9.6)	13 (4.8)	

*ASA* American Society of Anesthesiologists, *NACT* neoadjuvant chemotherapy, *PNI* physical status, prognostic nutritional index, *KPS* Karnofsky Performance Status Scale, *2D* two-field dissection, *2D+*, two-field+ dissection

^†^Numbers in parentheses are the percentages. ^‡^The location of the tumor in the thoracic esophagus

∗∗*P* < 0.01

The tumor was staged based on the 8th edition of the International Union Against Cancer /American Joint Committee on Cancer staging system. The PNI was defined as albumin concentration (g/L) + 5 × total lymphocyte count (10^9/L).

### Neoadjuvant chemotherapy

In our study, NACT was applied to patients with locally advanced (preoperative clinical TNM stage was cT1b-cT2 N+ or cT3-cT4a, any N) esophageal cancer which may have a low R0 resection rate. Regimen of docetaxel plus cisplatin was performed for patients. Patients were given docetaxel at a dose of 75 mg/m^2^ and cisplatin at a dose of 75 mg/m^2^ day 1, q21d per 2 cycles. After 2 cycles of NACT, tumor stage and efficacy of treatment were evaluated with cervical, chest, and abdomen computed tomography scan, upper digestive tract angiography, and ultrasonic gastroscopy for each patient. During chemotherapy, blood routine examination, and liver and kidney functions were reviewed. Furthermore, the degree of bone marrow suppression and liver and kidney function damage were evaluated. Esophagectomy was performed 4–6 weeks after the last chemotherapy cycle.

All the patients were reevaluated for target lesions after 2 cycles of chemotherapy according to the Response Evaluation Criteria in Solid Tumors (RECIST). The clinical efficacy was evaluated as follows: complete response (CR), partial response (PR), progressive disease (PD), or stable disease (SD). After 2 cycles of treatment, patients evaluated as CR\PR\SD underwent surgical treatment, and if the efficacy was evaluated as PD, the treatment strategy was changed.

### Operative procedures

All operations were performed by one experienced thoracic surgeon. All the patients received the same standard surgical procedure and perioperative management protocol. Esophagectomy with two-field lymph node dissection (D2) was a standard procedure; wide two-field+ lymph node dissection (D2+) was considered when the tumor was located in the upper or middle thoracic segment. D2+ lymph node dissection was defined as D2 lymph node dissection and bilateral lower cervical (under the inferior thyroid artery) paraoesophageal lymph nodes (no. 101) dissection.

Linear staples were used for esophagus cut off and gastric tube formation. The gastric sleeve atresia margin was embedded in the seromuscular layer by using absorbable sutures. Circular-stapled anastomat (COVIDIEN™ EEA™, America) was used to end-to-side anastomose the gastric tube and esophagus. Absorbable sutures were used to strengthen the anastomosis.

### ERAS protocols

Our ERAS protocol (Table 3) included 20 primary interventions in the preoperative, intraoperative, and postoperative periods, and the protocols were applied to all the patients. Our protocols are consistent with the basic idea behind the guideline published by Low et al. [10].

**Table 3:** ERAS protocol

Period	Intervention
Preoperative	1. Preadmission counseling
	2. Quit smoking and drinking 2 weeks before surgery
	3. Preoperative visit and evaluation
	4. Preoperative nutritional support
	5. Respiratory tract management
	6. No prolonged fasting
	7. No routine mechanical bowel preparation
Intraoperative	8. Antibiotic prophylaxis
	9. General anesthesia combined with erector spinae plane block (ESPB)
	10. The monitoring of anesthetic depth
	11. Maintaining normothermia
	12. Intraoperative infusion and circulatory system management
Postoperative	13. Postoperative pain management
	14. Postoperative nausea and vomiting prevention
	15. Early removal of urinary catheter
	16. Early ambulation
	17. Postoperative nutritional support
	18. Venous thromboembolism prophylaxis
	19. Early removal of thoracic drainage tube
	20. Patient education before hospital discharge

*EOF* early oral feeding, *ERAS* enhanced recovery after surgery, *ESPB* erector spinae plane block, *MBP* mechanical bowel preparation, *NS* normal saline, *POD* postoperative day

According to our ERAS protocols, patients with severe nutritional risk (>10% weight loss within 6 months; Pain score >5; body mass index (BMI)<18.5; serum albumin <30 g/L) received enteral nutrition supportive treatment. Preoperative respiratory tract management was performed for all patients.

Overnight fasting was changed, the patient received 800mL of 12.5% carbohydrate drinks 10h before surgery. During the operation, patients were administered general anesthesia combined with erector spinae plane (ESP) block. Ropivacaine hydrochloride (150 mg) was used for unilateral ESP block.

Patient-controlled intravenous analgesia (sufentanil citrate injection 0.1 mg + lappaconitine hydrobromide injection of 16 mg diluted with normal saline to 100 mL, 2 mL/h) was used for pain control until postoperative day (POD) 2–3. To maintain the patient’s central temperature at 36°C or above, a heated mattress and air conditioner were used. Prophylactic antibiotic helped reduce the incidence of postoperative infection. Prophylactic medication included both aerobic and anaerobic bacteria; the infusion were completed 30–60 min before the skin incision. If the operation duration was >3 h or estimated blood loss was >1000 mL, the antibiotics were reused once. On POD1, patients were allowed to lie in half position or move in the bed for an appropriate duration of time following surgery, did not need pillow removal for 6 h, and had ambulation. On POD2 patients were allowed to walk for about 10 min, twice a day, morning and afternoon. Then gradually increase the duration of walking.

Enteral nutrition support was combined with early oral feeding (EOF) in our protocols. The patient was provided 500 mL of 5% glucose and sodium chloride through the nasoduodenal tube within 24 h after surgery. Enteral nutrient emulsion (500 mL) was pumped through the enteral feeding pump at a constant speed from the nasointestinal tube (40–50 mL/h) on POD2 if the patient had stable vital signs. Oral feeding was allowed on POD2 or POD3. On the first day of oral feeding, only drinking water is allowed for the patients. Then, full liquid diet and semi-liquid diet (pureed foods or soft foods) were allowed successively. Full diet was allowed before patient discharged from the hospital.

The feeding was gradually increased to the full caloric requirement based on the total calories of 30 kcal/ (kg·d) under patient’s tolerance, and the enteral nutrition was gradually replaced by oral feeding. The insufficiencies in liquid and calories in the initial oral feeding stage were replenished through venous or enteral nutrition support. The parenteral nutrition was completely stopped on POD3 if there were no postoperative complications, but the nasointestinal tube still on working.

### Statistical analyses

In this study, SPSS version 26.0 (IBM Corp., Armonk, NY, USA) was used for statistical analysis. The patient characteristics in NACT group were compared with those in the non-NACT group. For comparison of categorical variables, we used Pearson chi-square or Fisher’s exact tests, whereas Wilcoxon test/Mann-Whitney *U* test or Student’s *t*-test was used for continuous variables regarding descriptive statistics, frequency, and percentage and *P* values were used for categorical variables’ description. Continuous variables are reported as mean (standard deviation), median (range), and *P* values. Two-sided P values of <0.05 were considered statistically significant.

## Results

### Patient characteristics

Patient characteristics for the two group are shown in Tables 2 and 4. There was no significant difference in gender, age, smoking and drinking history, comorbidities, KPS, and ASA status between NACT group and non-NACT group. Regarding surgical procedure, lymphadenectomy and estimated blood loss also showed no significant differences between the two groups. However, there was a significant difference in PNI (*P*<.001), operation duration (*P*=0.003), and clinical TNM stage (*P*<.001). In this study, a total of 34 patients did not have well treatment compliance during the treatment process, such as not accepting early oral feeding, not accepting early ambulation, etc. After discussion, we recognized that these patients were not suitable for continuing to implement ERAS protocols, so these patients were dropped out from protocols. And the overall compliance rate with the 20 main ERAS elements was 89.66% and 90.64%, respectively in the NACT and non-NACT groups.

**Table 4:** Clinical and pathological TNM stage

Stage	NACT (*n*=52)		Non-NACT (*n*=271)		*P*
c TNM stage^†^					<.001**
	I	0 (0)	I	60 (22.1)	
	II	11 (21.2)	II	148 (54.5)	
	III	28 (53.8)	III‡	52 (19.2)	
	IVa	13 (25.0)	IVa‡	11 (4.1)	
p TNM stage^†^	ypTNM		pTNM		
	I	18 (34.6)	Tis	3 (1.1)	
	II	20 (38.5)	Ia	20 (7.4)	
	IIIa	3 (5.8)	Ib	30 (11.1)	
	IIIb	7 (13.5)	IIa	33 (12.2)	
	IVa	4 (7.7)	IIb	73 (26.9.)	
			IIIa	45 (16.6)	
			IIIb	55 (20.3)	
			IVa	12 (4.4)	

*c TNM* clinical TNM, *NACT* neoadjuvant chemotherapy, *p TNM* pathological TNM

^†^Numbers in parentheses are the percentages. ^‡^Patient refused neoadjuvant chemotherapy

∗∗*P* < 0.01

### Clinical efficacy

In the NACT group, there was a total of 18 cases with a CR (5 cases with a pathologic CR), 29 cases with a PR, and 5 cases with SD. The rate of R0 resection (94.2% vs. 97.0% *P*=0.543) was not statistically significant in both groups. In the NACT group, most patients had advanced clinical stage (c TNM stage III and IVa accounted for 78.8%, while non-NACT group was 23.3%). The ypTNM stage of NACT group and pTNM stage of non-NACT group are shown in Table 4.

#### Short-term outcomes and pathological TNM stage

Table 5 demonstrates the short-term outcomes for both the NACT and non-NACT groups. There was no significant difference in length of stay (*P*=0.424), first bowel movement after the surgery (*P*=0.560), and the time of chest tube removal (*P*=0.238) between the two groups. There was a significant difference in the number of thoracic lymph nodes harvested (*P*=0.039), but the abdominal lymph nodes harvested was similar in both two group (*P*=0.528).

**Table 5:** Clinical short-term outcomes in the two groups

	NACT (***n***=52)	Non-NACT (***n***=271)	***P***
LOS, d	7.90±1.312	7.71±1.63	0.424
First bowel movement, d	2.92±0.71	3.01±0.99	0.560
Chest tube removal, d	5.37±1.46	5.13±1.29	0.238
Postoperative complications^†^	11 (21.2)	56 (20.7)	0.936
No. of total lymph nodes harvested	23.42±4.25	22.52±4.71	0.198
No. of thoracic lymph nodes harvested	15.46±3.83	14.07±4.52	0.039*
No. of abdominal lymph nodes harvested	7.96±2.73	8.44±3.36	0.331
R0 resection^†^	49 (94.2)	263 (97.0)	0.543
R1 resection^†^	0 (0)	0 (0)	
R2 resection^†^	3 (5.8)	8 (3.0)	

*LOS* length of stay, *NACT* neoadjuvant chemotherapy

^†^Numbers in parentheses are the percentages

∗*P* < 0.05

The severity grade and frequency of complications between the two groups are shown in Table 6. When comparing the NACT group with non-NACT group, the total postoperative complications rate did not show statistical difference (*P*=0.936). In addition, the rate of readmission within 30 days (*P*=0.587) and mortality in hospital (*P*>0.99) is also similar in the two groups. The complication severity grade is also similar in the two groups. Meanwhile, some frequent complications, such as postoperative atelectasis or pneumonia, surgical site infection, chylothorax, and anastomosis leakage, also had a similar incidence rate between the two groups (*P*>0.99).

**Table 6:** Postoperative complications

		NACT group (***n***=52)	Non-NACT group (***n***=271)	***P***
Morbidity		11 (21.2)	56 (20.7)	0.936
Readmission within 30 days		1 (1.9)	4 (1.5)	0.587
Mortality in hospital		0 (0)	2 (0.7)	>0.99
Severity grade of complications^†^	Grade I	4 (7.7)	9 (3.3)	0.705
	Grade II	2 (3.8)	17 (6.3)	
	Grade III	4 (7.7)	21 (7.7)	
	Grade IV	1 (1.9)	7 (2.6)	
	Grade V	0	2 (0.7)	
Frequent complications	Postoperative atelectasis or pneumonia	2 (3.8)	9 (3.3)	
	surgical site infection	0 (0)	4 (1.6)	
	Chylothorax	1 (1.9)	3 (1.1)	
	Anastomosis leakage	4 (7.7)	19 (7.0)	
	Vocal cord paralysis	4 (7.7)	6 (2.2)	

Numbers in parentheses are the percentages

*NACT* neoadjuvant chemotherapy

^†^Clavien-Dindo classification of the severity of surgical complications

However, there was a trend showing that the NACT group may have had a higher rate of vocal cord paralysis (*P* =0.097) compared to the non-NACT group.

## Discussion

ERAS has been implemented clinically for many years and accepted by a large number of surgeons, including urological, gastrointestinal, and gynecological surgeons [11–13]. There is only a limited amount of research about the implementation of ERAS for esophageal cancer patients at present. Particularly, there is no discussion about the feasibility of ERAS implemented in esophageal cancer patients who underwent NACT. In this retrospective study, our data proved that ERAS for NACT group patients are safe and feasible, although preoperative PNI and cTNM stage were significantly worse in the NACT group.

NACT followed by esophagectomy for treatment of ESCC has been widely accepted. Although chemotherapy may have some side effects such as leukopenia, previous studies have shown that NACT followed by esophagectomy is a feasible and safe, and there was no increase in postoperative complications and hospital length of stay after surgery [7, 8]. However, NACT may lead to necrosis and fibrosis, which can increase the surgical duration and intraoperative blood loss [6]. This was a little different from our study. In our study, NACT group patients had a longer operating time, yet there were no significant differences in intraoperative bleeding compared with the non-NACT group. The number of lymph nodes harvested was also similar for the two group. Meanwhile, Nomoto et al. [7] showed that NACT followed by esophagectomy did not increase the operation time and intraoperative blood loss.

Previous studies had suggested that the implementation of ERAS protocol in minimally invasive McKeown esophagectomy was safe and feasible [8]. In a randomized controlled trial about locally advanced gastric cancer, the author demonstrated that patients who received NACT can benefit from ERAS similarly to patients who were not administered NACT [14]. Our study showed that there were no significant differences between the two groups in postoperative complications or readmission, and that the hospital LOS after surgery was also similar between the two groups.

The overall complication rate was similar between the NACT group and non-NACT group in our study (22.1% vs. 21.0%). This was similar to a previous study [6], where they had demonstrated that NACT did not increase postoperative complications. However, we seem to have a lower postoperative complication rate compared with Ma et al. study (22.1% vs. 31.6%, 21.0% vs. 29.9%, respectively) [6]. In addition, the severity grade of complications was also similar in the two groups. Frequency of complications analysis showed that the NACT group had a higher rate in vocal cord paralysis, which may be explained by the fact that most patients in the NACT group had a locally advanced stage(c TNM stage ≥III) ESCC. There was no significant difference in LOS and the time of chest tube removal between the two groups. We demonstrated that ERAS for patients with NACT was also feasible and safe.

EOF is likely a crucial element of ERAS protocols for esophagectomy and an important contributor to the decrease in the postoperative LOS. Berkelmans et al. [15] showed EOF promotes recovery of gastrointestinal function without increasing the incidence and severity of postoperative complications. In Sun’s study [16], patients who underwent esophagectomy were allowed to eat a regular diet on POD1. They demonstrated that patients in the EOF group had a quicker recovery of bowel function and improved short-term quality of life without increased postoperative complications. However, some studies had shown that direct oral feeding following esophagectomy may increase the rate of anastomotic leakage and aspiration pneumonia [17, 18]. In our study, we adopted a more conservative feeding time. All patients included in this study received EOF and it was allowed on POD2 or POD3. Our results demonstrated that EOF for patients who received NACT were also safe, which did not increase the rate of anastomotic leakage and aspiration pneumonia. Meanwhile, it is worth considering whether we can implement a more aggressive EOF time.

The major purpose of NACT is to increase the rate of R0 resection through tumor down-staging for locally advanced cancer patients. In our study, there was no significant difference in R0 resection rate between the two groups, which is consistent with a previous study [8]. However, the NACT group had significantly more patients in the latter stages than the non-NACT group (*P* < 0.001, Table 4). In this case, it is reasonable to assume that NACT could lead to tumor down-staging.

A trend showed that NACT group may have had a high number of lymph nodes harvested compared with non-NACT group, especially in thoracic lymph nodes dissection. However, there was no statistical difference (*P*>0.05), which was different from previous studies [6–8]. A previous study showed that the location of lymph nodes might play a more important role than the number of lymph nodes harvested in esophagectomy, especially in the dissection of bilateral recurrent laryngeal nerve lymph nodes [19].

This study had some limitations. This was a retrospective study, and the disadvantages of such a study are that the patients enrolled were not randomly assigned and selection bias was unavoidable. Although a considerable number of patients with locally advanced stage refused neoadjuvant therapy and requested surgical treatment, the NACT group still contained more advanced cases than the non-NACT group. The sample size of the study was small, and it was a single-center study. Thus, further validation is necessary with multi-institution studies, which should include high-volume institutions. In addition, our study only included the patients who underwent neoadjuvant chemotherapy. Patients who underwent neoadjuvant radiotherapy and immunotherapy were not included. Therefore, the safety and feasibility of ERAS implemented in these patients remains to be further studied.

Neoadjuvant therapy plays an increasingly important role in patients with esophageal cancer, and ERAS will be accepted by more surgeons [20]. There will also be a trend to implement ERAS protocols for patients receiving neoadjuvant therapy. ERAS is not a specific standard nor invariable; thus, ERAS protocols should be improved constantly with the development of new approaches, methods, materials, technology, and equipment.

## Conclusions

In conclusion, in this study, we compared the short-term surgical outcomes in esophageal cancer patients who underwent NACT and non-NACT patients. The postoperative complications rate, postoperative hospital LOS, and readmission rate were similar in the two group. This study proved that ERAS protocols seemed to be safe and feasible for patients who received NACT before esophagectomy.

## Data Availability

The datasets used or analyzed during the current study are available from the corresponding author on reasonable request.

## Abbreviations

ERAS: Enhanced recovery after surgery
NACT: Neoadjuvant chemotherapy
ESCC: Esophageal squamous cell carcinoma
KPS: Karnofsky performance status scale
PNI: Prognostic nutritional index
ASA: American Society of Anesthesiologists
CR: Complete response
PR: Partial response
PD: Progressive disease
SD: Stable disease
BMI: Body mass index
ESP: Erector spinae plane
POD: Postoperative day
EOF: Early oral feeding
LOS: Length of stay
